# Greening Austrian social service and healthcare non-profits

**DOI:** 10.1016/j.heliyon.2023.e23767

**Published:** 2023-12-24

**Authors:** Philumena Bauer, Dorothea Greiling

**Affiliations:** Institute of Management Accounting, Johannes Kepler University, JKU Business School, Austria

**Keywords:** CSRD compliance, Environmental matters, Management controls, Institutionalism, Nonprofit-organizations, Interviews

## Abstract

The climate crisis requires the systematic integration of environmental matters into the management practices of companies across all sectors. The 2022 Corporate Sustainability Reporting Directive (CSRD) has created a need in many large social service and healthcare non-profits (SSHC–NPOs) within the European Union to extend the integration of environmental matters by 2025. While there is plenty of research on environment management practices of large for-profit enterprises, the research on environmental matters in SSHC-NPOs, which gain their legitimacy from social value creation, has been neglected. This study examines how large Austrian SSHC-NPOs are preparing for the environmental requirements set by the CSRD. The integration of environmental considerations into their core strategy, sustainable management control practices, and non-financial reporting poses a significant challenge. To evaluate the status of integration of environmental matters, the paper uses two sequential stage models based on institutional theory. The study is based on data from interviews with 21 Austrian SSHC-NPOs. The findings reveal that the integration of environmental matters is at an early stage, driven by a pragmatic approach with a strong emphasis on social and financial concerns. Cultural controls take precedence in management control practices, while administrative and cybernetic controls lag behind. Environmental reporting does not meet CSRD requirements, and the studied SSHC-NPOs aim for minimal compliance only, when CSRD comes into force in 2025. Additionally, it highlights that these organizations do not conform to the sequential stages proposed by two institutionalist stage models, emphasizing the role of the SSHC sector's context in shaping their behavior and practices.

## 1 Introduction

The climate crisis is one of the grand societal challenges. At all levels of governance, nation states must take far-reaching measures to counteract the already dramatic effects of the climate crisis. Austrian social service and healthcare (SSHC)-nonprofit-organizations (NPOs), on which the empirical part of the paper focuses, have to deal to an increasing extent with the detrimental effects of global warming in their fields of services. For example, the climate crisis has led to a significant increase in climate-induced migration, poverty, and social injustice. Not only in Austria, the climate crisis has led to an increase in heatwave days, which have negatively impacted the main beneficiary groups of SSHC-NPOs (children, the elderly and people with health issues). SSHC-NPOs are confronted with increasing ecological risks in their management activities and increasing expectations of resource-providing stakeholders to play a more proactive role in the green transformation processes. Tackling the climate crisis is an essential part of the societal mandate of SSHC-NPOs for a more just and fair society.

In line with the United Nations (UN) Agenda 2030 and the 2015 UN Paris Agreement, the European Union (EU) has intensified its coercive pressures towards a green transformation. One of the most recent steps taken by the EU Parliament is the adoption of the Corporate Sustainability Reporting Directive (CSRD) in November 2022. From 2025 onwards, the CSRD will also apply to SSHC-NPOs that are private law corporations in the (legal) form of a (non-profit) limited liability company or a (non-profit) public limited company. They will be subject to the CSRD if they meet two out of the three following criteria: at least 250 employees, a turnover of at least EUR 20 million, or a balance sheet total of at least EUR 40 million [1]. The CSRD creates a uniform framework for the disclosure of ESG (environmental, social, governance) matters. ESG-reporting requires governance structures for sustainable matters, policies and objectives, as well as a description of the main risks for companies in terms of sustainable activities. CSRD's dual focus on advancing external non-financial reporting and internal strategic and operative sustainability management activities is directed at reducing greenwashing by focusing not only on the talk level, i.e., non-financial reporting but also on the action level, i.e., sustainability strategy and sustainability management control practices (SMCP) [2]. SMCP represent a set of mechanisms and instruments that organizations use to monitor, measure and regulate their environmental, social and economic impacts. These mechanisms and instruments are crucial for the implementation of sustainable and responsible business practices [3,2].

While there is an extensive body of literature focusing on non-financial reporting and SMCP of for-profit and state-owned enterprises [e.g., [4], [5], [6], [7], [8], [9], [10]], the research on the integration of environmental matters into management control (MC) practices in SSHC-NPOs is at a very early stage (see section 2). Prior research on sustainability matters in SSHC-NPOs has mainly addressed social value creation [e.g., [11], [12], [13], [14], [15]] and financial matters [e.g., [16], [17], [18]]. This is not surprising as the social purpose of SSHC-NPOs plays a critical role in upholding organizational legitimacy. Financial matters have gained importance for SSHC-NPOs since the introduction of New Public Management (NPM) reforms in the late 1980s. For social service providing NPOs in corporatist welfare systems such as Austria, Germany, the Netherlands and the Benelux states [13], NPM reforms resulted in the loss of the privileged positions in welfare state arrangements between the service providing NPOs and the state. Reasons for this are the introduction of quasi-market elements, competitive tendering for short-term service provision contracts, public funding cuts and excessive reporting obligations towards public sector funders [13]. As a consequence of NPM, the financial risk for providing SSHC-services has been transferred from public funders to the service-providers. In corporatist, liberal and social welfare states alike, SSHC-NPOs have adopted business-style management practices to increase their managerial professionalism, and to become more entrepreneurial [19,13,20]. Service-providing NPOs often face the accusation of pursuing financial goals at the expense of their social missions when they adopt corporate management practices. The justification needs to deal with the accusations of mission drift towards legitimacy-creating stakeholders. This has increased significantly over the last ten years, even though no conclusive research (findings) on the performance reducing effects of mission drift has been established yet [19,20].

Compared to the academic body of literature on social and financial sustainability matters of SSHC-NPOs, the call for systematic integration of environmental aspects into the strategies, SMCP and non-financial reporting of SSHC-NPOs is much more recent. In this context, the paper addresses how large Austrian SSHC-NPOs are preparing for the increased integration of environmental matters in preparation for the mandatory implementation of the CSRD. The significance of environmental matters in SSHC-NPOs is unclear, as the organizational identity of SSHC-NPOs is linked to their social mission. This would justify the conclusion that environmental matters have a lower priority. The counter-argument is that SSHC-NPOs should actively contribute to reducing the negative impacts of climate change in line with their societal mandate. The main drivers for this are the increased expectations of stakeholders and the increasing demands on the services of SSHC-NPOs to address the negative impacts of climate change in times when public funding is decreasing and legislative pressures is increasing.

So far, NPOs and non-governmental organizations (NGOs) are portrayed by some authors as slow implementers regarding voluntary sustainability reporting [e.g., [21], [22], [23], [24]]. How that changes with the CSRD is unclear. In order to avoid the accusation of symbolic actions or greenwashing, it is necessary to move beyond mere non-financial reporting by integrating sustainable matters into an NPO's strategy and daily business [25]. SMCP play a central role in the promotion and realization of sustainable and responsible business practices [3,2]. While there is a growing body of literature on SMCP in general [e.g., [2], [25], [26]], SMCP in NPOs [e.g., [27], [28], [29]] and environmental management practices in NPOs [e.g., [30], [31]] have not received sufficient attention. Against this background, the paper addresses the following research question:

RQ: How well are Austrian SSHC-NPOs prepared to comply with the CSRD requirements regarding environmental matters?

a) How is the environmental dimension already implemented in their strategy and management controls and what steps are taken to improve the integration of the environmental dimension?
b) How are Austrian SSHC-NPOs preparing themselves to improve their reporting on environmental matters?

The subsequent sections are organized as follows. To answer the RQs, section 2 provides a brief overview of the existing literature on SMCP and environmental sustainability in NPOs as well as the conceptual and theoretical background for our study. Theory-wise, the paper draws on authors who use (neo-)institutionalism. To assess which level of environmental integration SSHC-NPOs are at, the paper uses two dynamic models, namely Shabana et al.'s [32] three-stage model and Gond et al.'s [3] three forms of sustainability integration in MC practices. Section 3 focuses on the methodology applied and provides an overview of the 21 Austrian SSHC-NPOs. According to current statistical data, the Austrian NPO sector accounts for 6 % of all employees in Austria. Among the various fields of activities within the Austrian NPO sector, SSHC-NPOs are employers of 54,4 % of the paid workforce in the Austrian NPO sector and generate 58 % of the economic value added of Austrian NPOs [33]. Among Austrian NPOs, SSHC-NPOs are the ones that are impacted the most by the CSRD, primarily due to their legal forms and size. The empirical results regarding the significance of environmental matters and their integration into strategic and operative management practices are presented in section 4. This is followed by a discussion of the results in section 5 which presents the answers to the research questions and an evaluation of the development stages of the integration of environmental matters in the focused SSHC-NPOs. The conclusions, limitations and areas for further research are presented in section 6.

With the focus of the paper being on the integration of environmental matters into the sustainability management practices of Austrian SSHC-NPOs, the paper contributes to the least addressed sustainability management dimension. The paper provides empirical insight regarding the assigned significance of environmental matters in comparison to social and financial matters. Furthermore, the paper investigates the extent to which the integration of environmental matters is implemented at the instrumental level, especially in non-financial reporting and SMCP. To the best of our knowledge, this paper is the first, to assess the readiness of SSHC-NPOs for the forthcoming EU CSRD concerning environmental matters in a corporatist welfare state system.

## 2 Conceptual and theoretical background

### 2.1 Conceptual background

There are several approaches which highlight the different MC types within management control systems (MCS) [e.g., [34], [35], [36]]. The most holistic one is the approach by Malmi and Brown [37,38], which places a special emphasis on the package idea of MC instruments and a wide range of informal (e.g., values, code of ethics) as well as formal control (e.g., key performance indicators (KPI), long and short term planning, bonus plans) [34, Table 1]. The package idea consists of five types of controls: cultural, planning, cybernetic, reward and compensation, and administrative. These controls must be integrated holistically to be effective as a package [34]. This framework is the one which has been often adopted when discussing the interplay between sustainability reporting and MC and the specific design of sustainability management control systems (SMCS) [[2], [4], [10], [39], Table 1].

**Table 1:** MCS and SMCS [2,34].

Control type	Management Control Systems [34]	Sustainability Management Control Systems [2]
*Cultural Controls*	Leverage cultural norms, values, clans, and beliefs to influence employee behavior and advance organizational objectives. Cultural Controls shape the organizational culture through mission, vision, and values.	Operationalizing social and environmental initiatives can intrinsically motivate employees to become more engaged in social and environmental goals.
*Planning*	Organizations employ processes and systems to set goals, devise strategies, allocate resources, and monitor performance. These processes distinguish between long-range planning and action planning.	Incorporation of short- and long-term sustainability goals into local and central planning processes. Communicating sustainability through goals provides meaningful direction and can reduce employee resistance to it.
*Cybernetic Controls*	Encompass various elements such as budgets, financial and non-financial measurement systems, and hybrid systems. Non-financial measurement systems may include audits, certification, or greenhouse gas accounting	To measure SD, corporate performance management, reporting, balanced scorecard, accounting, individual performance management and budgeting would be an option.
*Reward and Compens-ation*	To motivate employees, organizations use rewards like monetary incentives, bonuses, promotions, and recognition, aligning them with goals and performance achievements.	No differentiation is made regarding the control type reward and compensation. Incentives for achieving environmental goals are not widespread.
*Administra-tive Controls*	Include governance structure, organisation structure, policies, and procedures. Administrative controls enforce rules, policies, procedures, and hierarchical authority	Top management commitment creates awareness and vision for SD goals. New structures and visions require support through training, communication, feedback, employee engagement, cross-functional teams, and resource allocation

With the specific focus on organizational values within the cultural controls, the Malmi and Brown [34] framework is particularly suitable for SSHC-NPOs, where the social value orientations are of greater importance than in for-profit enterprises. The main objective of for-profit organizations is to generate profits, and social responsibility activities are often part of a strategy to reduce reputational risk and therefore have a serving function for financial value creation. As outlined above, social purpose is paramount to the organizational legitimacy of SSHC-NPOs with their mandate to create social value for society as well as for their clients and patients.

Regarding the academic debate on SMCP in NPOs, Daub et al. [29] concluded that there is hardly any research on the integration of corporate social responsibility or corporate sustainability into the management systems and processes of NPOs. Since 2014, only very few papers have been published which refer explicitly to SMCP and environmental sustainability in NPOs. If one takes a look at the existing literature on SMCP and environmental sustainability in NPOs and NGOs, it becomes apparent that the focus of the papers has been on selective SMCP in NPOs and NGOs [[27], [40], [41],^42^,^43^,^44^,^45^,^46^,^47^,^15^,^48^]. The main topics of the research on sustainability management in general are:

• *Partnerships and collaborations* [27,40,42,43,45,46,48]: These articles show, how NPOs and NGOs promote sustainability through strategic partnerships and collaborations. By collaborating with different entities, resources are pooled, impact is amplified and innovation is encouraged. These alliances strengthen advocacy, mitigate risk and contribute to long-term viability by diversifying funding sources and building organizational capacity.
• *Organizational structures and culture* [46,47]: The findings of these studies indicate that the organizational structure of an NGO should be carefully designed in order to achieve effectiveness and sustainability [46,47]. Furthermore, organizational culture significantly impacts sustainability [47]. In addition, a high employee retention rate and a stable workforce promote sustainability [44].
• *Strategic planning* [27,46,15]: Strategic planning guides NPOs to efficiently allocate resources, foster long-term impact, and align actions with sustainable development goals [27,46,15]. Weerawardena et al. [15] emphasized the relationship between sustainability and strategy and claimed that the creation of a sustainable organisation is closely linked to the strategic direction of the NGO.

Furthermore, there is hardly any academic research on environmental sustainability in NPOs and NGOs. Previous research has only dealt with the topics of:

• *Environmental orientation in general* [[49], [50], [51], [52], [53]]: These articles deal with the topic of environmental sustainability in NGOs in general.
• *Environmental management systems* [30,31]: RT White et al. [31] examined the implementation of the eco-management and audit scheme (EMAS) in an NPO. It is shown that by implementing EMAS, the organisation was able to identify operational improvements and make significant efforts to improve its environmental performance, reducing the carbon footprint per year. Chakraborty & Roy [30] investigated the implementation of environmental accounting (EMA), which provides combined monetary and physical information aimed at sustainable development, pollution prevention, and cleaner production methods.

If one evaluates these results from the perspective of Malmi and Brown's [34] package concept, it quickly becomes clear that there is a lack of a holistic view of SMCP. Only individual forms of controls are addressed. Furthermore, to the best of our knowledge, this research is the first, to assess the readiness of SSHC-NPOs for the EU CSRD concerning environmental matters, or how SSHC-NPOs are preparing themselves for the integration of environmental matters into their strategy and non-financial reporting.

### 2.2 Theoretical background

Research gaps exist in terms of evaluating the readiness of SSHC-NPOs to incorporate environmental concerns into MC practices and non-financial reporting. To address this gap, the paper employs institutional theory, in particular the three-stage model of Shabana et al. [32], which identifies a dominant isomorphism for each stage that shapes the non-financial reporting of organizations. Shabana's framework serves as a valuable tool to assess the progress of SSHC-NPOs in their adoption of non-financial reporting [see e.g., [54], [55]] but is also suitable for integrating sustainability matters into MC, as the implementation status of SMCP can also be categorized according to the three levels.

For extending the evaluation of the progress SSHC-NPOs made in integrating environmental matters into their SMCP, this paper additionally draws on the three dimensions of integration of sustainable matters by Gond et al. [3]. By focusing on the three levels of integration, Gond et al. [3] complement the three-level model of Shabana et al. [32] by focusing on the extent of socio-technical levels of integration of SMCP and MC practices [e.g., [56], [57]], or as Gond et al. [3, p. 2012] put it: “the thick interface between both types of management controls”.

Both classifications distinguish three levels of integration. Table 2 provides an overview of the linkage of the three-phase model of Shabana et al. [32] with Gond et al. [3].

**Table 2:** Linkage of Shabana et al. [32] with Gond et al. [3].

Stage 1	Stage 2	Stage 3	
Coercive isomorphism	Normative isomorphism	Mimetic isomorphism	Shabana et al. [32]
Technical integration	Cognitive integration	Organizational integration	Gond et al. [3]

In the first stage of Shabana et al. [32] coercive isomorphism, i.e., new regulatory requirements or societal expectations are the dominant pressure. Coercive isomorphism leads to defensive and selective non-financial reporting. Firms fail to meet stakeholder expectations and at most, narrow the stakeholder expectation gap [32]. Transferring that reasoning onto SMCP, one could argue that only very selective SMCP are implemented during this stage. According to Gond et al. [3] technical integration is characterized by the fact that only selective environmental MCS are implemented. Although there might be several environmental KPI in place, there is no systematic approach to steering and monitoring environmental matters. A broader set of MC practices is in place which does not focus on SMCP [3]. As a consequence, two parallel systems exist. Sustainable matters are primarily dealt with outside the MC function [3].

In the second phase, normative isomorphism drives non-financial reporting and SMCP resulting in highly ambitious approaches regarding sustainability matters. All three dimensions of sustainability are essential to the mission, strategy and managerial practices of SSHC-NPOs. Normative isomorphism leads to the broad application of SMCP [32]. At this stage, the development of shared understanding requires cognitive integration [3]. Opportunities for discussion are created between people who bring different ways of thinking, mentalities, and practical viewpoints regarding environmental matters. The outcome of these processes should be reflected in a shared understanding of sustainability objectives and strategy [3].

In the third phase, mimetic isomorphism dominates. Approaches of other organizations are imitated if they are perceived to be successful, and the benefits of sustainable management begin to outweigh the costs [32]. Organizations take a pragmatic stance toward the integration of environmental matters into their strategy and SMCP. According to Gond et al. [3], appropriate governance and organizational structures are fully established and organizational actors with varying levels of professional socialization and from different hierarchical level differences have developed joint SMCP and non-financial reporting. The integration of environmental matters into MC and strategy should not only be seen as something an organisation possesses, but also as something that people do [3].

## 3 Methodology and sample

A total of 22 expert interviews were conducted with 29 top management representatives from 21 Austrian SSHC-NPOs since autumn 2022. The interview guideline contained open-ended questions and covered the following thematic blocks: interviewees' understanding of sustainability, operational and strategic management of environmental matters, the status of SMCP based on Malmi and Brown [34] and Lueg and Radlach [2] and the steps towards a non-financial reporting in line with the CSRD requirements. The interviews lasted between 70 and 120 minutes and were conducted in Austria, partly in person and partly online via Zoom. The interviews were recorded, transcribed, and then deductively and inductively coded using the MAXQDA 2022 software. Table 3 provides an overview of the SSHC-NPOs included.

**Table 3:** Overview of SSHC-NPOs

Name	Organisation type	Number of employees	Required to report according to CSRD
Caritas der Diözese St. Pölten	Church-related social economy organizations	2,500 full-time and 841 voluntary employees	X
Caritas der Erzdiözese Wien		5,400 full-time and 4541 voluntary employees	X
Caritas Oberösterreich		3,200 employees 1100 voluntary employees	X
Caritas Sozialis Privatstiftung		900 full-time and 300 voluntary employees	
Evangelische Diakoniewerk Gallneukirchen		9,900 full-time and 2,500 voluntary employees	X (for the hospitals)
Stiftung Liebenau		7,758 full-time employees	X
Geriatrisches Gesundheitszentrum der Stadt Graz	Secular socioeconomic organizations	660 full-time employees	X
Hilfswerk Niederösterreich Betriebs GmbH		3,000 full-time and 2,800 voluntary employees	X
Jugend am Werk Steiermark GmbH		1,300 full-time employees	X
Kuratorium Wiener Pensionisten Wohnhäuser		4,800 full-time employees	
Österreichische Jungarbeiterbewegung ÖJAB		750 full-time employees	
Österreichisches Rotes Kreuz		9,668 full-time and 74,804 voluntary employees	
Volkshilfe Oberösterreich		1,900 full-time employees	
Volkshilfe Wien gemeinnützige Betriebs-GmbH		1,476 full-time and 400 voluntary employees	X
Wien Work - integrative Betriebe und AusbildungsgmbH		700 full-time employees	X
Barmherzige Brüder Österreich	Hospitals	64,000 full-time and 29,000 voluntary employees	X
Franziskus Spital GmbH		660 full-time employees	X
Niederösterreichisches Landesgesundheitsagentur		2,700 full-time employees	X
Oberösterreichische Gesundheitsholding GmbH		15,300 full-time employees	X
Vinzenzgruppe Kranhausbeteiligungs- und Management GmbH		9,689 full-time employees	X
Vorarlberger Krankenhausbetriebsgesellschaft m.b.h.		4,991 full-time employees	X

The overview of the sample shows that our interview partners are important actors in Austrian SSHC-NPOs. The sample was selected to comprise secular SS-NPOs, church-affiliated SS-NPOs, and HC-NPOs. The HC-NPOs category is comprised of sizable religious HC-NPOs and state-owned hospitals. The latter were included because they derive their legitimacy from their public mission mandates, which prioritize non-profit objectives over for-profit ones.

The methodology section inherently possesses certain limitations, particularly in relation to sample size, study period, and the geographical emphasis on Austrian SSHC-NPOs (refer to section 6 for further details). While we believe that the depth of our interviews reached a satisfactory saturation point, it is essential to acknowledge potential criticisms related to the selection of the interviewees, given that the empirical part of the paper is based only on insights from 21 NPOs. Size-wise the included SSHC-NPOs are important actors. The number of employees ranges from 660 to 64,000. Only five SSHC-NPOs in our sample have less than 1,000 employees. All are well above the CSRD threshold of 250 employees [1].

## 4 Results

### 4.1 Strategic relevance of sustainability

#### 4.1.1 Understanding of sustainability

In the interview section focusing on the organizational and personal understanding of sustainability, most of the interview time was dedicated by the interviewees to the *environmental dimension* of sustainability. Some interviewees provided examples of the efforts they have taken to reduce their personal CO_2_ footprint and the role their children play in questioning the behavior of their parents’ generation. When asked about their understanding of sustainability and its relationship to their efforts to achieve the goals of the UN 2030 Agenda, a third of organizations reported their organizational commitment to social and climate justice (I1; I2; I3; I4; I7; I8; I14; I21; I22).

The *social dimension* of sustainability was classified by the interviewees as their organizational core. In SSHC-NPOs, history and organisational values play an important role in presenting their social commitments. In all three types of SSHC-NPOs, the importance of the social dimension was emphasized. This was underlined by their current social purpose, their organizational self-image, and the fact that they are SSHC-NPOs whose primary objective is to provide a wide range of social and healthcare services to the public, rather than to generate profits. The interviewees also stressed that their organizations are committed to providing safe, high-quality client and patient care within their tight financial constraints. In particular, interviewees from HC-NPOs stressed their contribution to regional development.

In all SSHC-NPOs, the *financial dimension* is also very important. The economic efficiency of service provision is given high priority (I2; I3; I5; I6; I7; I8; I9; I11; I13; I15; I16; I20). All top-level executives emphasized that their organizations function as businesses and, therefore, must adhere to business logic. As enterprises, they must generate sufficient revenue to prevent losses. This does not exclude SSHC-NPOs from cross-subsidizing some services internally, but the overall profit and loss statement must cover costs. Unlike private for-profit social and health service providers, profit maximization is not an objective of the sampled SSHC-NPOs.

In comparison, the *environmental dimension* of sustainability has only gained priority in recent years [e.g., [[58], [59]]] and is not yet on equal footing with the social and financial dimensions. Depending on the world-view, this is justified by the increasingly urgent obligation to use the earth's resources in a much more ecologically sustainable manner (I3; I9; I10; I11; I13; I15) and the responsibility for the planet or the responsibility for creation (I2; I3; I4; I13; I15; I16; I17; I18; I19; I20; I22). For example: *"Sustainability, of course, means much more than just climate protection. When I think of biodiversity and many other issues, like the protection of the oceans [ …], but my personal motivation has always been especially all that has to do with climate protection“*(I15). CEOs from Catholic hospitals and church-related social economy organizations stressed the importance of the papal encyclical Laudato Si for improving their environmental orientation.

A recurring topic was the trade-offs between financial, environmental and social sustainability. Conflicts between the three dimensions of sustainability are resolved at the expense of the environmental dimension. The interviewees emphasized that the commitment to environmental sustainability must be profitable (I5; I7; I10; I18; I19; I20) which is shown pars pro toto in the following quote: *“You have to be able to make a business out of it. We are not profit-oriented, but we cannot operate in areas that are permanently in deficit."* (I7).

#### 4.1.2 Environmental engagement as part of the sustainability strategy

Environmental sustainability is already included into the strategic orientation of nine SSHC-NPOs (I4; I5; I6; I7; I9; I13; I14; I15; I120). With varying prioritization, another eight organizations have already named environmental sustainability as a strategic field (I4; I6; I7; I9; I13; I14; I15; I120). Across the eight SSHC-NPOs, there are significant variations. Environmental and climate protection issues were mentioned by the interviewees as a strategic priority. The importance assigned to environmental and climate change actions varied in the eight SSHC-NPOs from one in four to one in 25 strategic priorities. A prominent strategic objective for a third of the SSHC-NPOs is to be climate neutral in 2030, as the following quote shows: „ *[w]e have all collectively resolved, as an organisation itself, to be climate neutral or CO*_*2*_ *neutral in 2030[ …]"* (I4).

In three organizations, environmental sustainability is considered a cross-cutting issue that cannot be reduced to a strategic field of action (I1; I13; I16), as the following quote illustrates:

*"[s]ustainability plays the highest role in our strategy because it is an underlying inner attitude that is relevant in all decisions."* (I13).

In nine organizations, environmental sustainability is not yet an integral part of the corporate strategy (I2; I8; I10; I11; I12; I17; I18; I19; I22) but some interviewees stressed the importance of integrating environmental objectives in the near future, as the following quote illustrates: *"[t]hat is our weak point, so to speak. I think we have a well-developed attitude on the subject in the hierarchy and an institutional opinion. And where it comes to actually formulating a sustainability strategy across all areas in our organization, I would say we have the headline and table of contents"* (I19). Only one interviewee opposed the integration of ecological sustainability into the organizational strategy: *“I want to be number one in care, I want to be number one in nursing and care services, I want to be the best employer in the social sector."* (I10).

**Fig. 1:**
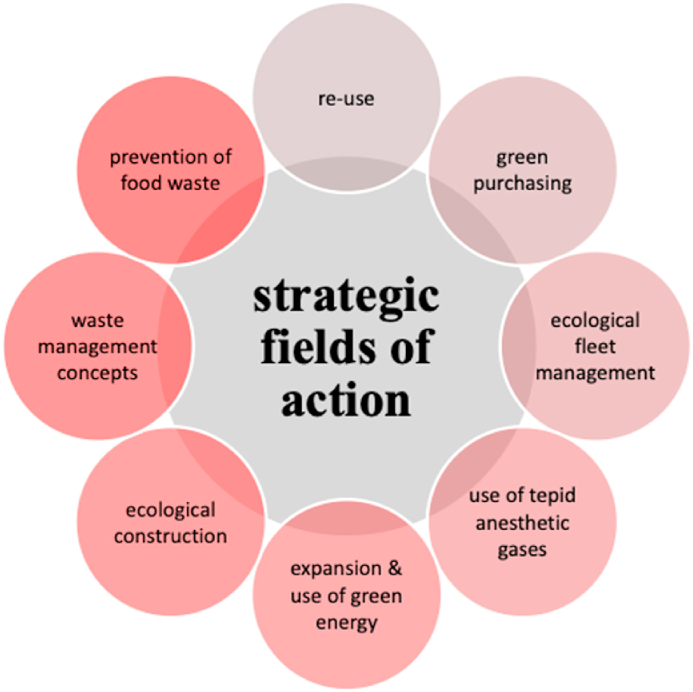
Strategic fields of action.

With regard to *strategic fields of action* (Fig. 1) for the environmental transformation, references were made to the reduction of the carbon footprint through an appropriate mobility strategy for employees (I1; I4; I5; I6; I8; I12; I14; I20), more ecological fleet management (I10; I12), the expansion of the use of green energy and green aesthetic gases (I1; I2; I4; I5; I7; I8; I10; I13; I14; I16; I17; I19; I20). Also mentioned were green buildings (I8; I11; I13; I14; I19), the intensification of green purchasing (I2; I4; I7; I8; I12; I14; I17; I19), the existence of appropriate waste management concepts and the already existing approaches to avoid food waste (I1; I4; I6; I8; I12; I13). Another topic was the aspect of "re-use", in which interviewees, especially those who operate hospitals and care facilities, addressed the limits of re-use. Some interviewees stressed that re-use starts with green procurement. Greening the organisation was described by numerous interviewees as a highly challenging topic.

### 4.2 Management controls

**Cultural Controls:** Concerning the five MC types by Malmi and Brown [34], the interviewees assigned the highest relevance to intensifying the environmental orientation in their cultural controls. Interviewees stressed the importance of their organizational culture for being a societal responsible organization, as the following quotes illustrate: "*[c]orporate culture [ ….], leading by example and authenticity."* (I16); *"You don't need so much else if you have the appropriate organizational culture.“* (I11).

The majority of interviewees stressed the importance of persons within their organizations who are leading by example when it comes to sustainability matters. Some interviewees linked that to a strong intrinsic motivation and highlighted the importance of their NPO's values. They are crucial for SSHC-NPOs as they guide decision-making, shape organizational culture, and align actions with their mission (I1; I2; I3; I4; I6; I7; I8; I12; I16; I20). Concerning environmental matters a few interviewees mentioned the importance of leading by example, because it establishes norms, inspires and motivates less committed employees, and reinforces desired behaviors (I7; I8; I14; I15). Interviewees are well aware of the essential role of an organization-wide communication strategy regarding environmental matters (I1; I2; I3; I4; I8; I11). Organization-wide awareness building, proactively communicating green transformation projects, and embedding the importance of green transformation in personnel development programs are all steps to an organization-wide roll-out of environmental matters.

**Planning:** Regarding long-term planning, many church-related SS-NPOs and HC-NPOs with a recent EMAS certification stressed their ambitions for a net-zero footprint. About a third of the SSHC-NPOs aim to be climate-neutral by 2030 as the following quote illustrates: *„We have all collectively resolved, as an organisation itself, to be climate neutral or CO*_*2*_ *neutral in 2030 [ …]"* (I4). Several church-related NPOs indicated that if compensation for unavoidable CO_2_ emissions is necessary, they intend to invest in their projects in the Global South because their own engagement in the Global South will be of more value than other compensation initiatives. Collecting donations for climate protection projects in the Global South is also a part of today's donor-fundraising of church-related SS-NPOs.

The integration of environmental concerns into short-term planning appears to be in its infancy, as the connection between the two was generally discussed in a vague manner. A minority of interviews mentioned internal annual target agreements (I5; I6; I9; I14) with the middle and lower management level but in a more general way. Only in one EMAS-certificated HC-organization, environmental targets are systematically integrated into the yearly performance evaluations of division managers.

**Cybernetic Controls:** The interviews stressed that SSHC-NPOs already have a broad set of financial, performance, and quality indicators. SSHC-NPOs are in an early stage when it comes to environmental KPI, as the following quote illustrates: *„That I say I have a key figure that shows me whether I am in the green zone, whether I have to readjust something. Frankly we are not there yet. […]“* (I11). In one-third of the SSHC-NPOs, selective KPI are implemented for measuring energy efficiency (I3; I8; I9; I15; I17; I20) and waste management-related concerns (I1; I4; I6; I8; I12; I13). Those SSHC-NPOs with a recent environmental certification (EMAS: I9; I15, other eco-certificates: I1; I4; I7; I19) or who have voluntarily implemented a greenhouse gas accounting covering scope 1 and scope 2 (I3; I8; I9; I15; I17; I20) are better prepared in some areas. Two SSHC-NPOs (I14; I17) which use the balanced scorecard as a strategic management tool stressed that they intend to incorporate environmental matters into their balanced scorecards in the future. In two other organizations, a specific environmental cockpit is in place to monitor their environmental performance. The integration of environmental risks into risk management is still in its infancy (I2; I3; I4; I8; I11; I14). Due to the adverse effects of the war in Ukraine on energy delivery security and energy prices, three NPOs had established a risk management task force for energy safety risks at the time of the interviews (I8; I9; I11).

**Reward and Compensation:** Financial incentives for employees were rejected by all interviewed CEOs due to a decremental crowding out effect as expressed in the following quote: *"Incentives are generally difficult in NPOs because, as I said, people are very strongly intrinsically motivated. And then often such a reward or a monetary incentive system is even counterproductive."* (I2). The interviewees stressed that their organizations offer a variety of voluntary social benefits for environmental sustainability, such as support for environmental tickets for local public transport (I3; I16; I17; I18), electric bicycles and to a much lesser extent electric cars (I2; I7; I10; I12; I16; I17; I19). Activities are also offered to promote health in the workplace (I7; I10; I12; I16; I17). Furthermore, two SSHC-NPOs are offering bonuses to individuals who do not use parking spaces (I15; I16). A fairly common measure is financial support for the purchase of electric bicycles. (Covered) bicycle parking spaces are being created to incentivize bicycle commuting (I2; I3; I12; I16; I18; I20) in addition to providing free bicycle repair services (I2; I3; I12; I16; I18; I19).

Six interviewees (I12; I14; I15; I16; I18; I20) emphasized their well-established idea management system and their culture of appreciation. Rewards range from non-monetary benefits to a budget for implementing award-winning measures. The focus on environmental matters in idea management activities was generally vague, with a few exceptions related to food waste reduction or the use of green anesthesia.

**Administrative Controls:** The CEO or top management board is ultimately responsible for organizational governance for sustainability matters (I2; I5; I6; I7; I9; I11; I12; I13; I14; I15; I16; I18; I19; I20). Table 4 displays organizational governance structures in hierarchical lower management levels.

**Table 4:** Organizational governance.

Environmental management officers and sustainability managers	Reporting directly to the board
Special sustainability and innovation staff units	Reporting directly to the CEO
Management teams below the top level in faith-based and secular social economy organizations	Involved in sustainable management issues and green transformation issues
Division managers	Responsible for ecological issues
Special project task forces or working groups	Promote the topic of sustainability and environment in general and advance climate protection activities by focusing on green energy transformation, greening the mobility of employees and a greener housekeeping

Two organizations are in the process of establishing a climate protection officer (I4; I7), as the following quote illustrates: *“[a]nd I say as it looks at the moment, we will have someone as sustainability or climate protection manager also in the future, who will also have the responsibility for sustainability reporting."* (I6).

Regarding policies and procedures, a few SSHC-NPOs mentioned that they have anti-corruption, anti-fraud guidelines, information technology and health and safety policies, guidelines for ethical investments as well as regional and green procurement and green building. Environmental policies and procedures were the least addressed ones.

Table 5provides an overview of the key findings regarding the five MC types of Malmi and Brown [34].

**Table 5:** Key findings.

**Cultural Controls**			
• Mission statement • Founding mission • Intrinsic motivation • Organisational values • Organizational wide awareness building for environmental mattes • Communication			
**Planning** *Strategic Planning* • Reduction of carbon footprint • Reduction of CO_2_ emissions • Climate-neutral by 2030 *Action Planning* • Internal annual target agreements	**Cybernetic Controls** *Financial measurements* • Annual reporting • Cost center evaluations • Liquidity and monthly reports • Budgets for climate protection measures • Greenhouse gas accounting *Non-financial measurements* • Sustainability Reporting • Environmental statements • Energy reports *Hybrid measurements* • Balanced Scorecard • Consumption, quality and energy indicators *Environmental management systems and concepts* • EMAS certifications • Eco-certificates *Planned* • Integration of sustainable matters into the risk management		Reward and Compensation *Voluntary social benefits* • Workplace health promotion activities • Support for green mobility *Idea and innovation initiatives*
**Administrative Controls**			
*Organizational responsibility structures* • CEO or the management board • Environmental management officers • Sustainability managers *Division managers for environmental matters* • Facility management • Procurement department • Head of the catering division in one SS-NPO *Special sustainability and innovation staff units* • Sustainability staff units • Diversity and organizational value units • Sustainability and innovation staff units *Special project task forces or working groups* *Management teams*		*Policies, Guidelines and Programs* • Guidelines for regional and green procurement • Environmental policies and procedures • Additional guidelines: Anti-corruption, Anti-fraud guidelines, IT- and health and safety policies, ethical investments	

### 4.3 Non-financial reporting

**Fig. 2:**
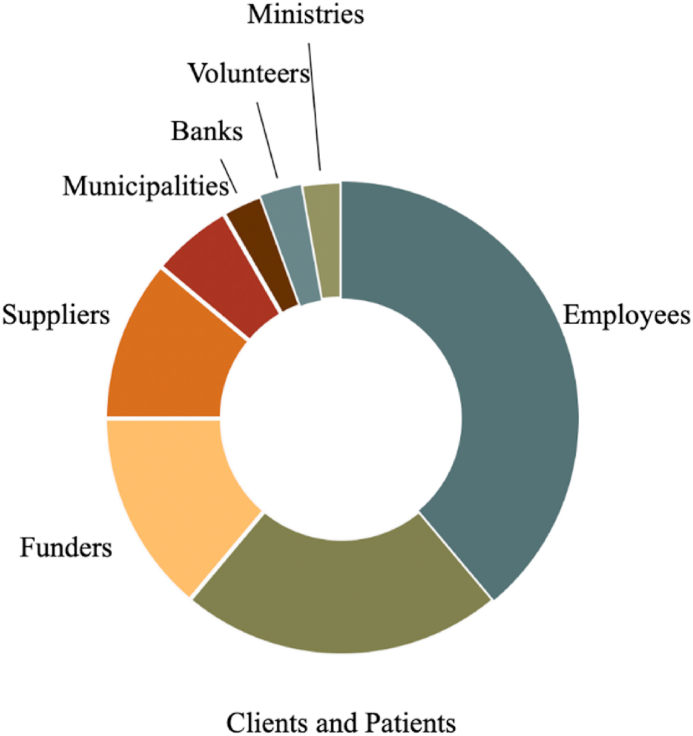
Key stakeholder groups.

As Fig. 2 illustrates, and as discussed in the section on cultural controls, employees play a critical role in communicating environmental issues (Fig. 2). Followed by clients and patients, funders, suppliers, municipalities, banks, volunteers and ministries.

For internal stakeholders, environmental matters are communicated through newsletters (I1; I2; I8; I9; I11; I16; I18), the intranet (I4; I6; I9), information mails (I7) and online platforms (I12). Environmental matters are also part of the information provided by the top management to the lower management levels regarding organizational strategic priorities (I4; I11; I18).

External stakeholder communication on environmental matters include website appearances (I6; I8; I9; I16), annual reports (I6; I9), poster campaigns (I12), and social media channels (I7; I12; I16). The integration of environmental criteria into the requirements of public funders is still very limited, with the exception of public funding earmarked for green building initiatives, programs for supporting a green energy transformation, or energy saving programs. Gaining access to the EU-sustainable finance fund was not addressed by the interviewees.

None of the SSHC-NPOs currently meet the advanced ESG-reporting requirements for environmental issues. The six SSHC-NPOs with existing greenhouse gas accounting (I3; I8; I9; I15; I17; I20) are in a slightly better starting position but even for these organizations, a fully-fledged ESG-reporting on environmental matters will be a challenge. The roadmaps presented in the interviews by those 16 SSHC-NPOs that have to publish an ESG-report (see Table 4) are very ambitious, considering the present status of environmental KPI. There is still a lot to do, as the quote illustrates. *"[I]t's a relative monster project […] you need an insane amount of numbers, data, facts […]. Not only energy figures but really quite a lot of other things as well […]."* (I1)

None of the interviewees made reporting on environmental matters their top priority, as the quote shows: *"[ …] I don't think it is the highest priority, even if I am honest, to produce the report."* (I4)*.* Other interviewees stressed that the organisation will be a lazy follower in implementing the CSRD.

## 5 Discussion

The awareness of the general relevance of the environmental dimension of sustainability is high within the focused SSHC-NPOs. Regarding our overall research question *„How well are Austrian SSHC-NPOs prepared to comply with the CSRD requirements regarding environmental matters?“,* the findings of our study show that all organizations still have a huge amount of work ahead regarding their environmental management (strategy, SMCP and ESG-reporting). Austrian SSHC-NPOs take a pragmatic approach towards environmental matters, incorporating them as much as economically feasible. If there are trade-offs between the environmental dimension and the social purpose, they are solved at the expense of the former. The way the interviewees referred to the trade-offs between the ecological dimension and the other two sustainability dimensions suggests a compartmentalized, non-integrated approach. Since NPM, SSHC-NPOs have already a tradition of choosing a compartmentalization approach. This strategic response is usually chosen to become more business-like, while not comprising their social mission identity [13,20].

Regarding sub-question one *„How is the environmental dimension already implemented in their strategy and management controls and what steps are taken to improve the integration of the environmental dimension?“*, the interviewed SSHC-NPOs are in an early stage of systematic integration of environmental sustainability into their strategy and SMCP. The interviewees stressed the crucial role of cultural controls. The analyzed SSHC-NPOs are in an early instrumental stage regarding planning, environmental cybernetic controls and administrative controls. While steps are taken to improve the inclusion of selective environmental commitments into long-term planning, the integration of environmental matters into short-term planning and therefore steering the operational practices is nearly non-existent. Linking environmental matters to financial rewards was opposed because of a decremental crowding-out of intrinsic motivation. Recalling the idea of MC as a package [34], the current implementation status of SMCP is far too selective to do justice to the idea of interlinking the various SMCP for the implementation of environmental matters. While the international literature review by Lueg and Radlach [2] showed cybernetic controls to be the first choice followed by administrative SMCP, our sample of SSHC-NPOs prioritize cultural controls. This is in line with their self-image as social value-driven organizations and the significance of the social dimension of sustainable matters [13]. The deliberate and selective adoption of SMCP while neglecting others becomes evident, highlighting that SSHC-NPOs are in a very early stage of organizational change process for integrating environmental matters.

Concerning sub-question two *„How are Austrian SSHC-NPOs preparing themselves to improve their reporting on environmental matters?“*, the findings show that those 16 SSHC-NPOs, which must publish an ESG-report in 2025, are in the early stages of their journey towards ESG-reporting. That aligns with prior literature which portrays NPOs as having a slow and highly selective approach to implementing non-financial reporting [21,22,23,24]. In line with that none of the interviewees identified ESG-reporting and therefore improving the reporting on environmental matters as their top priority. The findings also show that employees are the top communication addressees for environmental matters. The relaxed approach to environmental reporting by the 16 SSHC-NPOs, which must comply with CSRD in 2025, corresponds with the pragmatic approach towards the environmental dimension.

Based on an evaluation of the findings from the perspective of Shabana et al.'s [32] three-stage model, the coercive pressure to improve non-financial reporting on environmental matters was still very weak in the perception of our interviewees. This was the case regardless of whether they have to comply with the CSRD in 2025 or are voluntarily establishing non-financial reporting on environmental issues (e.g., in the form of greenhouse gas accounting, carbon footprints). The roadmap of what needs to be done to comply with the CSRD requirements is quite ambitious, given the short time frame before the publication of the first ESG-report in 2025.

More in line with the second stage of Shabana and co-authors [32] is, that nine SSHC-NPOs have already embedded environmental aspects in their strategy and, with one exception, the remaining SSHC-NPOs plan to improve the integration of environmental matters into their strategy. Additionally, the interviewees stressed the great importance of cultural controls in advancing their SMCP. That behavior is directed toward making the environmental dimension an essential part of the organizational values. As the CSRD was only based in 2022, mimetic isomorphism is the leased addressed one.

Recalling Gond et al. [3], SSHC-NPOs heavily rely on a cognitive integration. Therefore, they put not the technical integration of MC and sustainability management activities first, as suggested by Gond et al. [3]. The integration of environmental KPI is still in its early stages. Regarding the organisational integration of environmental matters, first steps are taken for adapting governance structures. Environmental matters are the least addresses once in the sustainable management policies and procedures. The importance attached to cognitive integration [3] and cultural controls [34] by the analyzed SSHC-NPOs allows two interpretations. First, the unfriendly one: Due to the rudimentary stage of integrating environmental matters into their strategy and the deplorable status of environmental KPI, one could conclude that most of the SSHC-NPOs have moved to the next talk level by emphasizing the need to first get the organizational strategy right and by acknowledging that green transformation requires a long organizational change process. Fitting with the next talk level is also the relaxed approach of the CSRD-affected SSHC-NPOs towards improving their non-financial reporting till 2025. In line with that is also the highly pragmatic approach towards advancing the environmental dimension only if that does not come at the costs of social and financial objectives.

The more friendly interpretation is that the SSHC-NPOs do the right thing by first aiming to integrate environmental matters into their organizational values and culture due to the fact that they are mission-driven organizations. The managers of the SSHC-NPOs stressed many times that the commitment towards the social dimension of sustainability or their societal mandates are the cornerstone for their existence, belief systems and organizational identity. That is a positive sign in the debate about mission-drifts of social service providing NPOs due to NPM reforms [13,20].

## 6 Conclusions

The paper contributes to the academic debate in the following ways: Empirically, by focusing on the status quo and the envisaged steps of SSHC-NPOs towards the CSRD, the paper addresses a timely and highly under-researched field. Austrian SSHC-NPOs have a wait-and-see attitude toward the coercive pressures of advancing their reporting on environmental matters. Furthermore, our findings indicate that there are no signs that environmental matters will be put on equal footing with the other two sustainability dimensions in the near future. Social and financial matters are prioritized at the cost of environmental matters. A highly pragmatic approach prevails regarding environmental matters. The study examines the integration level to which environmental aspects are considered at the instrumental level, particularly in non-financial reporting and SMCP. In particular, this study represents the first attempt to assess the preparation of SSHC-NPOs within a corporatist welfare state for the forthcoming EU CSRD with a focus on environmental concerns.

From a *theoretical point of view*, the paper indicates, that “doing things differently” also challenges the three stages by Shabana et al. [32] and Gond et al. [3]. The analyzed SSHC-NPOs have a clear preference for integrating the environmental dimension into their organizational culture while being far less willing to invest in elaborate environmental KPI. That also impacts the financial reporting side on environmental matters. The in-betweenness had to be expected, as the phases are ideal types, but the particular blend offered by SSHC-NPOs tentatively indicates that NPOs values or social mission orientation need to be taken into account as a significant context factor.

Concerning *practical implications*, the implementation of environmental MC and improving the non-financial reporting in SSHC-NPOs is crucial for several reasons. SSHC-NPOs heavily rely on public support and trust, necessitating the enhancement of their public image as responsible environmental stewards. By adopting environmental MC and communicating their environmental commitment, SSHC-NPOs could show their dedication to responsible practices which also plays a major role in employer branding. Implementing environmental MC would allow them to assess and monitor their activities, identify areas of concern and opportunities for cost savings, as well as implement strategies to reduce their negative impact on the environment. Recalling the Austrian government's commitment to Agenda 2030, the greening of their procurement practices will most likely come earlier than expected for SSHC-NPOs. Finance institutions will also increase pressure for sustainable finance in the near future.

This study has several limitations, especially with regard to sample size, study period, and the regional focus on Austrian SSHC-NPOs. The focus on large SSHC-NPOs allows us to gain insights into the adoption of environmental matters in the most important fields of the Austrian NPOs with respect number of employees, the economic value creation and the increase of employees in the past decade [33]. The drawback is that the results are not transferable to other fields of the NPO sector, such as advocacy organizations, cultural NPOs, or NPOs where the main actors are volunteers. Another caveat is that Austria is a corporatist welfare state and therefore the findings might be different from liberal welfare states or welfare states with a social-democratic tradition. Although we had the impression that the interview richness had reached a reasonable saturation, the selection of the interview partners can be criticized because our paper is based on 21 NPOs. We have so far done deductive and inductive coding and have not yet integrated a documentary analysis of all the publications of the 21 SSHC-NPOs addressing the environmental dimension. A next step for further research could be to look at how another corporatist welfare states, as the SSHC-NPOs are under the same green transformation pressures.

## Data availability statement

The data has not been deposited into a public available repository.

The authors do not have permission to share data.

## Ethics approval statement

All participants/patients provided informed consent to participate in the study. All participants/patients provided informed consent for the publication of their anonymized case details and images.

## CRediT authorship contribution statement

**Philumena Bauer:** Writing – review & editing, Writing – original draft, Visualization, Software, Project administration, Methodology, Investigation, Formal analysis, Data curation, Conceptualization. **Dorothea Greiling:** Writing – review & editing, Writing – original draft, Resources, Project administration, Methodology, Investigation, Formal analysis, Data curation, Conceptualization.

## Declaration of competing interest

The authors declare that they have no known competing financial interests or personal relationships that could have appeared to influence the work reported in this paper.
